# Supplementary material for: BCG vaccine protection from severe coronavirus disease 2019 (COVID-19)
Source: Proc Natl Acad Sci U S A. 2020 Jul 9;117(30):17720–6. doi: 10.1073/pnas.2008410117 (PMC7395502; doi:10.1073/pnas.2008410117)
Supplement: Supplementary File [file pnas.2008410117.sapp.pdf]

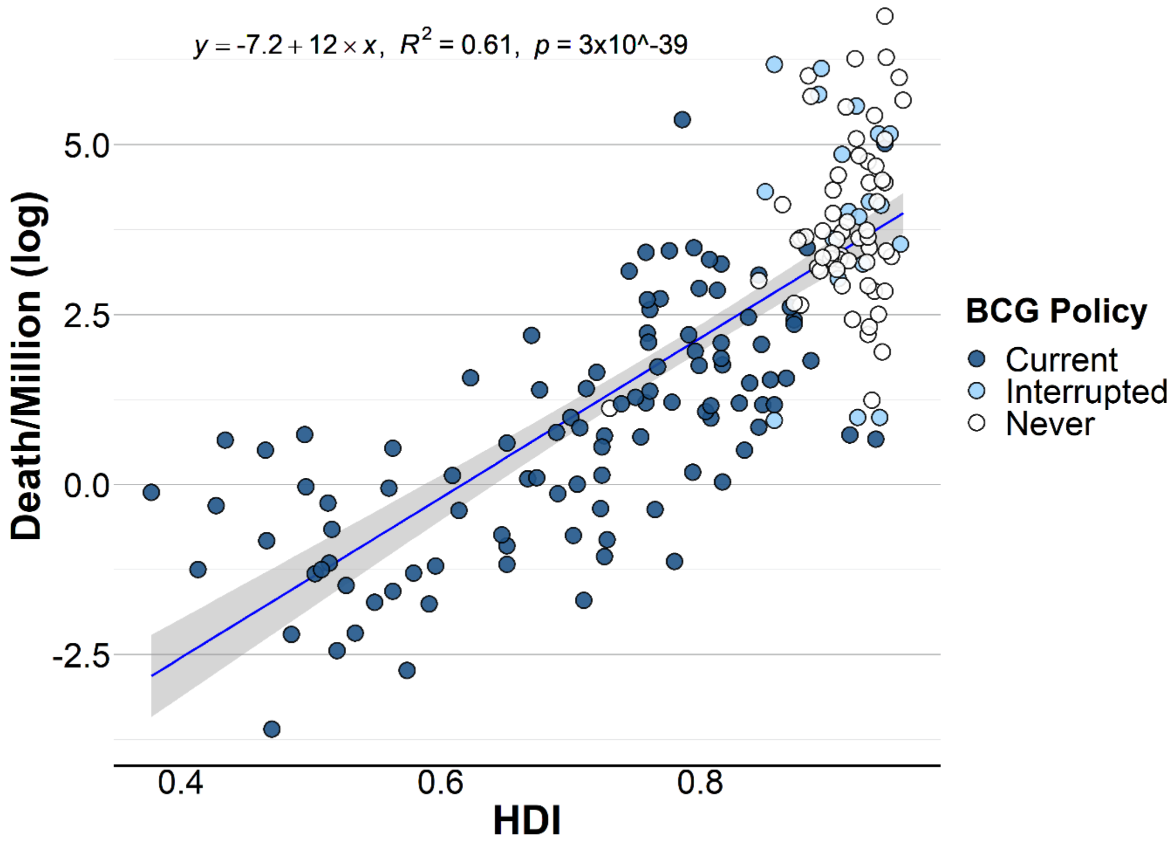

Figure S1. Relation between Human Development Index and COVID-19 deaths per million (log). BCG policy: current vaccination (dark blue), interrupted vaccination (blue), and never implemented BCG vaccination program (light blue). United States analyzed as states. Table S1 contains the summary of results between confounding variables and COVID-19 mortality.

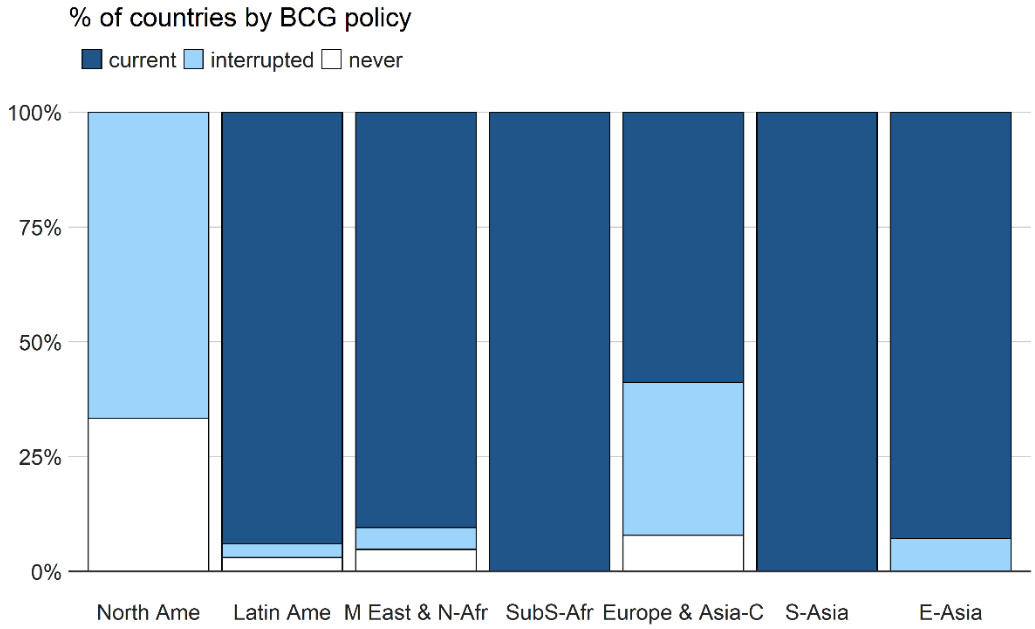

Figure S2. BCG vaccination policies by world region. Percentage of countries by region in terms of their BCG policy: current vaccination (dark blue), interrupted vaccination (blue), and never implemented BCG vaccination program (light blue). North Ame=North America; Latin Ame=Latin America; M East & N-Afr=Middle East and North Africa; SubS-Afr=Sub Sahara Africa; Europe & Asia-C=Europe and Asia Central; S-Asia=South Asia; E-Asia=East Asia. United States analyzed as a country.

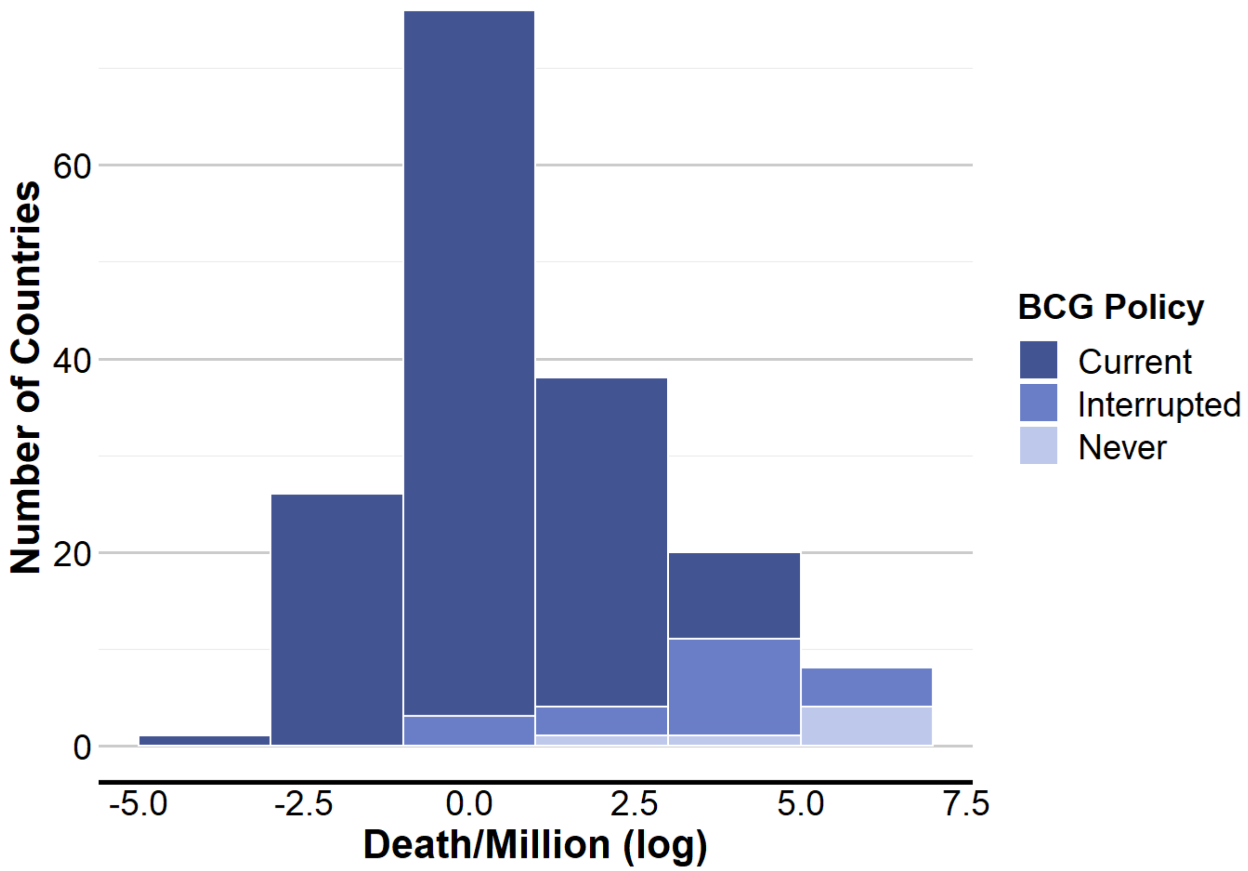

Figure S3. Histogram o BCG vaccination policies and COVID-19. BCG policy denoting current vaccination (dark blue), interrupted vaccination (blue), and never implemented BCG vaccination program (light blue) and (log) total deaths per country per 1 M inhabitants. United States analyzed as a country.

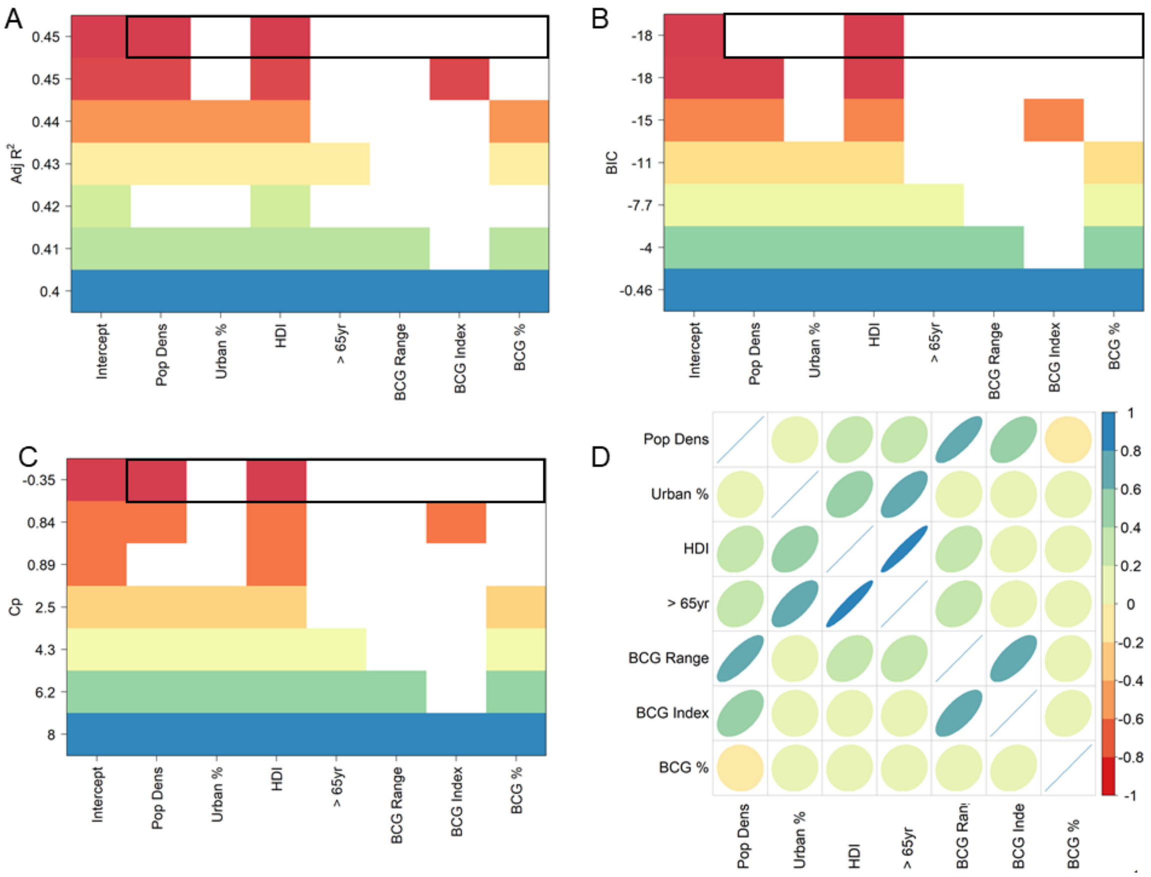

Figure S4. Multivariate model of COVID-19 mortality. We developed multiple linear regression models using (log) COVID-19 deaths accumulated during the first month of reported mortalities for all countries as dependent variable and the potential confounding variables and BCG data as the predictor variables. Statistic diagnostic metrics were used to determine the best variable combination and select the optimal model for COVID-19 death estimation. The regression model diagnostic procedure included the adjusted Bayesian information criterion (BIC) which measures model error adjusted by number of variables; adjusted r^2, which measures the proportion of variation in the dependent variable that is explained by the predictor variables adjusted by number of variables; and the Mallow's C_p, which measures model error accounting for sample size, and number and collinearity of predictors. **A.** The adjusted r^2 evaluation found that the best model should include two variables, population density and HDI (square). **B.** The BIC evaluation found HDI as the best variable for the model (square). **C.** The Mallow's C_p evaluation also found that the best model should include density and HDI (square). **D.** Correlogram denoting high (narrow ellipsoid) or none (broad ellipsoid and yellow color) correlation. All correlations were positive (ellipsoid direction to the right and blue color). The best variable combination was HDI and population density, which significantly predicted COVID-19 deaths/country/first month of mortality (r^2=0.49, $F(2,113)$=55.57, p<0.001). **HDI**: Human Development Index; **Pop Dens**: Population density; **Urban %**: Percentage urbanization; **>65 yr**: Population above >65 yrs (%); **BCG %**: Percentage of population vaccinated annually using BCG; **BCG Range**: Number of years of BCG vaccination program; **BCG Index**: Combination of percentage of population vaccinated annually using BCG and number of years of BCG vaccination program by country.

Supplementary Table S1. Correlation analyses between social variables and COVID-19 mortality. Analyses included the United States as a single country (**Coarse analysis (United States as a Country)**) and by state (**Coarse analysis (United States as states)**), because some of its states are larger than many European, Asian, and Latin American countries. Analyses were conducted globally before and after controlling for confounding variables (**Refined analysis (Controlling Confounding Variables)**). Mortality values are based on original numbers by country and corrected by the country's population and by the time of the epidemic to allow standardized evaluations. Significant analysis ($p<0.05$) are denoted in red. **DF**= degrees of freedom, $\mathbf{R^2}$= correlation coefficient, **AIC**=Akaike's information criteria. Note that association decreases considerably after accounting for controlling variables. Pop Dens: Population Density (inhabitants/km^2); Urban (%): Percentage of population in urban areas; HDI: Human Development Index; >65 years: Percentage of population that is >65 year-old.

Dependent Variable	Independent Variable	DF	R^2	P-Value	AIC
**Coarse analysis (United States as states)**					
Deaths/1 M (Total)	Pop Dens	1	0.001	0.588	2914.320
Days to 0.1 Death/1 M	Pop Dens	1	0.001	0.625	1279.182
Days to 1 Death/1 M	Pop Dens	1	0.007	0.304	1206.722
Deaths/day/1 M (mean)	Pop Dens	1	0.004	0.385	1056.207
Deaths/day/1 M (median)	Pop Dens	1	0.000	0.939	915.769
Deaths/day/1 M (max)	Pop Dens	1	0.029	0.015	1764.574
Deaths/1 M/week 3 (Total)	Pop Dens	1	0.012	0.142	1756.090
Deaths/1 M/week 3 (median)	Pop Dens	1	0.000	0.929	787.638
Deaths/1 M/week 3 (mean)	Pop Dens	1	0.012	0.142	1063.340
Deaths/1 M/week 3 (max)	Pop Dens	1	0.060	0.001	1451.154
Deaths/1 M/month 1 (Total)	Pop Dens	1	0.002	0.596	1499.382
Deaths/1 M/month 1 (median)	Pop Dens	1	0.001	0.735	511.885
Deaths/1 M/month 1 (mean)	Pop Dens	1	0.002	0.597	655.847
Deaths/1 M/month 1 (max)	Pop Dens	1	0.003	0.526	1075.932
Deaths/1 M log (Total)	Pop Dens	1	0.005	0.311	898.985
Deaths/day/1 M log (mean)	Pop Dens	1	0.007	0.249	872.667
Deaths/day/1 M log (median)	Pop Dens	1	0.006	0.402	472.296
Deaths/day/1 M log (max)	Pop Dens	1	0.016	0.073	836.233
Deaths/1 M/week 3 log (Total)	Pop Dens	1	0.012	0.176	678.003
Deaths/1 M/week 3 log (median)	Pop Dens	1	0.009	0.313	456.243
Deaths/1 M/week 3 log (mean)	Pop Dens	1	0.012	0.177	676.995
Deaths/1 M/week 3 log (max)	Pop Dens	1	0.026	0.043	646.020
Deaths/1 M/month 1 log (Total)	Pop Dens	1	0.001	0.788	532.055
Deaths/1 M/month 1 log (median)	Pop Dens	1	0.010	0.348	368.584
Deaths/1 M/month 1 log (mean)	Pop Dens	1	0.001	0.780	531.682
Deaths/1 M/month 1 log (max)	Pop Dens	1	0.000	0.831	507.402
Deaths/1 M (Total)	Urban (%)	1	0.099	0.000	2925.364

Days to 0.1 Death/1 M	Urban (%)	1	0.074	0.000	1309.837
Days to 1 Death/1 M	Urban (%)	1	0.000	0.783	1200.273
Deaths/day/1 M (mean)	Urban (%)	1	0.099	0.000	1040.405
Deaths/day/1 M (median)	Urban (%)	1	0.076	0.000	903.314
Deaths/day/1 M (max)	Urban (%)	1	0.070	0.000	1763.694
Deaths/1 M/week 3 (Total)	Urban (%)	1	0.065	0.001	1764.073
Deaths/1 M/week 3 (median)	Urban (%)	1	0.067	0.000	782.223
Deaths/1 M/week 3 (mean)	Urban (%)	1	0.065	0.001	1063.541
Deaths/1 M/week 3 (max)	Urban (%)	1	0.054	0.002	1466.704
Deaths/1 M/month 1 (Total)	Urban (%)	1	0.104	0.000	1508.242
Deaths/1 M/month 1 (median)	Urban (%)	1	0.065	0.004	510.036
Deaths/1 M/month 1 (mean)	Urban (%)	1	0.104	0.000	651.121
Deaths/1 M/month 1 (max)	Urban (%)	1	0.088	0.001	1080.245
Deaths/1 M log (Total)	Urban (%)	1	0.254	0.000	847.856
Deaths/day/1 M log (mean)	Urban (%)	1	0.231	0.000	829.093
Deaths/day/1 M log (median)	Urban (%)	1	0.153	0.000	453.241
Deaths/day/1 M log (max)	Urban (%)	1	0.218	0.000	795.271
Deaths/1 M/week 3 log (Total)	Urban (%)	1	0.238	0.000	652.232
Deaths/1 M/week 3 log (median)	Urban (%)	1	0.194	0.000	432.300
Deaths/1 M/week 3 log (mean)	Urban (%)	1	0.238	0.000	651.372
Deaths/1 M/week 3 log (max)	Urban (%)	1	0.231	0.000	621.102
Deaths/1 M/month 1 log (Total)	Urban (%)	1	0.248	0.000	515.794
Deaths/1 M/month 1 log (median)	Urban (%)	1	0.211	0.000	347.252
Deaths/1 M/month 1 log (mean)	Urban (%)	1	0.246	0.000	515.835
Deaths/1 M/month 1 log (max)	Urban (%)	1	0.250	0.000	487.056
Deaths/1 M (Total)	HDI	1	0.123	0.000	2659.118
Days to 0.1 Death/1 M	HDI	1	0.117	0.000	1212.673
Days to 1 Death/1 M	HDI	1	0.082	0.000	1124.599
Deaths/day/1 M (mean)	HDI	1	0.125	0.000	921.263
Deaths/day/1 M (median)	HDI	1	0.101	0.000	844.645
Deaths/day/1 M (max)	HDI	1	0.095	0.000	1537.169
Deaths/1 M/week 3 (Total)	HDI	1	0.108	0.000	1423.723
Deaths/1 M/week 3 (median)	HDI	1	0.107	0.000	729.857
Deaths/1 M/week 3 (mean)	HDI	1	0.108	0.000	773.760
Deaths/1 M/week 3 (max)	HDI	1	0.108	0.000	1005.717
Deaths/1 M/month 1 (Total)	HDI	1	0.114	0.000	1342.062
Deaths/1 M/month 1 (median)	HDI	1	0.080	0.002	479.930
Deaths/1 M/month 1 (mean)	HDI	1	0.114	0.000	546.087
Deaths/1 M/month 1 (max)	HDI	1	0.134	0.000	854.510
Deaths/1 M log (Total)	HDI	1	0.623	0.000	649.287
Deaths/day/1 M log (mean)	HDI	1	0.606	0.000	637.164
Deaths/day/1 M log (median)	HDI	1	0.434	0.000	381.678
Deaths/day/1 M log (max)	HDI	1	0.546	0.000	626.506

Deaths/1 M/week 3 log (Total)	HDI	1	0.505	0.000	527.312
Deaths/1 M/week 3 log (median)	HDI	1	0.514	0.000	344.079
Deaths/1 M/week 3 log (mean)	HDI	1	0.511	0.000	524.324
Deaths/1 M/week 3 log (max)	HDI	1	0.485	0.000	499.179
Deaths/1 M/month 1 log (Total)	HDI	1	0.538	0.000	422.392
Deaths/1 M/month 1 log (median)	HDI	1	0.503	0.000	284.851
Deaths/1 M/month 1 log (mean)	HDI	1	0.543	0.000	420.793
Deaths/1 M/month 1 log (max)	HDI	1	0.535	0.000	393.861
Deaths/1 M (Total)	>65 yrs	1	0.128	0.000	2708.633
Days to 0.1 Death/1 M	>65 yrs	1	0.046	0.003	1280.570
Days to 1 Death/1 M	>65 yrs	1	0.028	0.036	1150.943
Deaths/day/1 M (mean)	>65 yrs	1	0.109	0.000	935.356
Deaths/day/1 M (median)	>65 yrs	1	0.106	0.000	846.402
Deaths/day/1 M (max)	>65 yrs	1	0.060	0.001	1596.830
Deaths/1 M/week 3 (Total)	>65 yrs	1	0.089	0.000	1444.094
Deaths/1 M/week 3 (median)	>65 yrs	1	0.083	0.000	728.648
Deaths/1 M/week 3 (mean)	>65 yrs	1	0.089	0.000	763.002
Deaths/1 M/week 3 (max)	>65 yrs	1	0.099	0.000	983.317
Deaths/1 M/month 1 (Total)	>65 yrs	1	0.072	0.003	1370.980
Deaths/1 M/month 1 (median)	>65 yrs	1	0.054	0.010	449.527
Deaths/1 M/month 1 (mean)	>65 yrs	1	0.072	0.003	534.213
Deaths/1 M/month 1 (max)	>65 yrs	1	0.068	0.003	875.041
Deaths/1 M log (Total)	>65 yrs	1	0.520	0.000	731.014
Deaths/day/1 M log (mean)	>65 yrs	1	0.496	0.000	714.728
Deaths/day/1 M log (median)	>65 yrs	1	0.233	0.000	434.998
Deaths/day/1 M log (max)	>65 yrs	1	0.435	0.000	693.506
Deaths/1 M/week 3 log (Total)	>65 yrs	1	0.360	0.000	598.941
Deaths/1 M/week 3 log (median)	>65 yrs	1	0.337	0.000	403.255
Deaths/1 M/week 3 log (mean)	>65 yrs	1	0.362	0.000	597.436
Deaths/1 M/week 3 log (max)	>65 yrs	1	0.340	0.000	564.431
Deaths/1 M/month 1 log (Total)	>65 yrs	1	0.319	0.000	485.541
Deaths/1 M/month 1 log (median)	>65 yrs	1	0.290	0.000	329.855
Deaths/1 M/month 1 log (mean)	>65 yrs	1	0.320	0.000	485.194
Deaths/1 M/month 1 log (max)	>65 yrs	1	0.309	0.000	457.203
**Coarse analysis (United States as a Country)**					
Deaths/1 M (Total)	Pop Dens	1	0.001	0.655	2187.555
Days to 0.1 Death/1 M	Pop Dens	1	0.003	0.532	964.352
Days to 1 Death/1 M	Pop Dens	1	0.012	0.263	828.453
Deaths/day/1 M (mean)	Pop Dens	1	0.005	0.404	727.131
Deaths/day/1 M (median)	Pop Dens	1	0.001	0.725	605.678
Deaths/day/1 M (max)	Pop Dens	1	0.051	0.006	1247.601
Deaths/1 M/week 3 (Total)	Pop Dens	1	0.013	0.208	1265.946
Deaths/1 M/week 3 (median)	Pop Dens	1	0.001	0.667	450.147

Deaths/1 M/week 3 (mean)	Pop Dens	1	0.013	0.208	775.576
Deaths/1 M/week 3 (max)	Pop Dens	1	0.064	0.004	1064.879
Deaths/1 M/month 1 (Total)	Pop Dens	1	0.000	0.934	1065.788
Deaths/1 M/month 1 (median)	Pop Dens	1	0.001	0.767	326.697
Deaths/1 M/month 1 (mean)	Pop Dens	1	0.000	0.934	467.178
Deaths/1 M/month 1 (max)	Pop Dens	1	0.000	0.839	781.640
Deaths/1 M log (Total)	Pop Dens	1	0.010	0.237	656.683
Deaths/day/1 M log (mean)	Pop Dens	1	0.013	0.174	632.416
Deaths/day/1 M log (median)	Pop Dens	1	0.024	0.184	304.504
Deaths/day/1 M log (max)	Pop Dens	1	0.028	0.043	602.092
Deaths/1 M/week 3 log (Total)	Pop Dens	1	0.022	0.121	463.359
Deaths/1 M/week 3 log (median)	Pop Dens	1	0.018	0.256	287.331
Deaths/1 M/week 3 log (mean)	Pop Dens	1	0.022	0.121	463.359
Deaths/1 M/week 3 log (max)	Pop Dens	1	0.047	0.023	441.974
Deaths/1 M/month 1 log (Total)	Pop Dens	1	0.004	0.536	380.280
Deaths/1 M/month 1 log (median)	Pop Dens	1	0.019	0.284	242.927
Deaths/1 M/month 1 log (mean)	Pop Dens	1	0.004	0.536	380.280
Deaths/1 M/month 1 log (max)	Pop Dens	1	0.004	0.543	359.710
Deaths/1 M (Total)	Urban (%)	1	0.085	0.000	2217.583
Days to 0.1 Death/1 M	Urban (%)	1	0.066	0.002	995.903
Days to 1 Death/1 M	Urban (%)	1	0.000	0.967	829.731
Deaths/day/1 M (mean)	Urban (%)	1	0.082	0.000	722.986
Deaths/day/1 M (median)	Urban (%)	1	0.063	0.002	602.310
Deaths/day/1 M (max)	Urban (%)	1	0.056	0.004	1261.790
Deaths/1 M/week 3 (Total)	Urban (%)	1	0.054	0.008	1278.581
Deaths/1 M/week 3 (median)	Urban (%)	1	0.052	0.010	448.802
Deaths/1 M/week 3 (mean)	Urban (%)	1	0.054	0.008	780.428
Deaths/1 M/week 3 (max)	Urban (%)	1	0.052	0.010	1081.336
Deaths/1 M/month 1 (Total)	Urban (%)	1	0.094	0.003	1079.227
Deaths/1 M/month 1 (median)	Urban (%)	1	0.053	0.029	327.356
Deaths/1 M/month 1 (mean)	Urban (%)	1	0.094	0.003	467.011
Deaths/1 M/month 1 (max)	Urban (%)	1	0.073	0.010	790.637
Deaths/1 M log (Total)	Urban (%)	1	0.227	0.000	629.224
Deaths/day/1 M log (mean)	Urban (%)	1	0.201	0.000	611.427
Deaths/day/1 M log (median)	Urban (%)	1	0.167	0.000	292.774
Deaths/day/1 M log (max)	Urban (%)	1	0.184	0.000	584.360
Deaths/1 M/week 3 log (Total)	Urban (%)	1	0.255	0.000	446.726
Deaths/1 M/week 3 log (median)	Urban (%)	1	0.223	0.000	270.480
Deaths/1 M/week 3 log (mean)	Urban (%)	1	0.255	0.000	446.726
Deaths/1 M/week 3 log (max)	Urban (%)	1	0.242	0.000	427.325
Deaths/1 M/month 1 log (Total)	Urban (%)	1	0.234	0.000	373.296
Deaths/1 M/month 1 log (median)	Urban (%)	1	0.230	0.000	228.183
Deaths/1 M/month 1 log (mean)	Urban (%)	1	0.234	0.000	373.296

Deaths/1 M/month 1 log (max)	Urban (%)	1	0.234	0.000	349.578
Deaths/1 M (Total)	HDI	1	0.141	0.000	1910.680
Days to 0.1 Death/1 M	HDI	1	0.059	0.005	907.382
Days to 1 Death/1 M	HDI	1	0.015	0.225	763.142
Deaths/day/1 M (mean)	HDI	1	0.135	0.000	572.907
Deaths/day/1 M (median)	HDI	1	0.106	0.000	549.459
Deaths/day/1 M (max)	HDI	1	0.154	0.000	912.650
Deaths/1 M/week 3 (Total)	HDI	1	0.086	0.001	916.494
Deaths/1 M/week 3 (median)	HDI	1	0.095	0.001	412.015
Deaths/1 M/week 3 (mean)	HDI	1	0.086	0.001	465.043
Deaths/1 M/week 3 (max)	HDI	1	0.071	0.004	657.109
Deaths/1 M/month 1 (Total)	HDI	1	0.089	0.006	910.863
Deaths/1 M/month 1 (median)	HDI	1	0.053	0.038	306.016
Deaths/1 M/month 1 (mean)	HDI	1	0.089	0.006	353.066
Deaths/1 M/month 1 (max)	HDI	1	0.105	0.003	560.261
Deaths/1 M log (Total)	HDI	1	0.566	0.000	481.687
Deaths/day/1 M log (mean)	HDI	1	0.530	0.000	473.094
Deaths/day/1 M log (median)	HDI	1	0.379	0.000	249.801
Deaths/day/1 M log (max)	HDI	1	0.462	0.000	460.321
Deaths/1 M/week 3 log (Total)	HDI	1	0.430	0.000	361.348
Deaths/1 M/week 3 log (median)	HDI	1	0.438	0.000	221.609
Deaths/1 M/week 3 log (mean)	HDI	1	0.430	0.000	361.348
Deaths/1 M/week 3 log (max)	HDI	1	0.386	0.000	345.403
Deaths/1 M/month 1 log (Total)	HDI	1	0.469	0.000	308.905
Deaths/1 M/month 1 log (median)	HDI	1	0.457	0.000	190.375
Deaths/1 M/month 1 log (mean)	HDI	1	0.469	0.000	308.905
Deaths/1 M/month 1 log (max)	HDI	1	0.448	0.000	287.503
Deaths/1 M (Total)	>65 yrs	1	0.214	0.000	1956.813
Days to 0.1 Death/1 M	>65 yrs	1	0.016	0.135	978.532
Days to 1 Death/1 M	>65 yrs	1	0.006	0.443	797.325
Deaths/day/1 M (mean)	>65 yrs	1	0.217	0.000	530.521
Deaths/day/1 M (median)	>65 yrs	1	0.177	0.000	517.042
Deaths/day/1 M (max)	>65 yrs	1	0.203	0.000	898.579
Deaths/1 M/week 3 (Total)	>65 yrs	1	0.213	0.000	822.430
Deaths/1 M/week 3 (median)	>65 yrs	1	0.238	0.000	290.847
Deaths/1 M/week 3 (mean)	>65 yrs	1	0.213	0.000	335.952
Deaths/1 M/week 3 (max)	>65 yrs	1	0.172	0.000	530.350
Deaths/1 M/month 1 (Total)	>65 yrs	1	0.172	0.000	872.824
Deaths/1 M/month 1 (median)	>65 yrs	1	0.178	0.000	167.847
Deaths/1 M/month 1 (mean)	>65 yrs	1	0.172	0.000	274.213
Deaths/1 M/month 1 (max)	>65 yrs	1	0.142	0.000	527.099
Deaths/1 M log (Total)	>65 yrs	1	0.513	0.000	534.520
Deaths/day/1 M log (mean)	>65 yrs	1	0.478	0.000	520.365

Deaths/day/1 M log (median)	>65 yrs	1	0.279	0.000	274.859
Deaths/day/1 M log (max)	>65 yrs	1	0.412	0.000	499.242
Deaths/1 M/week 3 log (Total)	>65 yrs	1	0.351	0.000	403.386
Deaths/1 M/week 3 log (median)	>65 yrs	1	0.386	0.000	245.664
Deaths/1 M/week 3 log (mean)	>65 yrs	1	0.351	0.000	403.386
Deaths/1 M/week 3 log (max)	>65 yrs	1	0.310	0.000	381.382
Deaths/1 M/month 1 log (Total)	>65 yrs	1	0.332	0.000	344.149
Deaths/1 M/month 1 log (median)	>65 yrs	1	0.371	0.000	206.958
Deaths/1 M/month 1 log (mean)	>65 yrs	1	0.332	0.000	344.149
Deaths/1 M/month 1 log (max)	>65 yrs	1	0.321	0.000	318.651
**Refined analysis (Controlling Confounding Variables)**					
Deaths/1 M (Total)	Pop Dens	1	0.223	0.023	289.977
Days to 0.1 Death/1 M	Pop Dens	1	0.008	0.689	144.635
Days to 1 Death/1 M	Pop Dens	1	0.004	0.764	167.649
Deaths/day/1 M (mean)	Pop Dens	1	0.217	0.025	105.680
Deaths/day/1 M (median)	Pop Dens	1	0.195	0.035	107.871
Deaths/day/1 M (max)	Pop Dens	1	0.088	0.169	157.385
Deaths/1 M/week 3 (Total)	Pop Dens	1	0.035	0.403	165.624
Deaths/1 M/week 3 (median)	Pop Dens	1	0.053	0.305	73.388
Deaths/1 M/week 3 (mean)	Pop Dens	1	0.035	0.403	80.004
Deaths/1 M/week 3 (max)	Pop Dens	1	0.021	0.523	107.093
Deaths/1 M/month 1 (Total)	Pop Dens	1	0.068	0.268	214.614
Deaths/1 M/month 1 (median)	Pop Dens	1	0.055	0.318	52.227
Deaths/1 M/month 1 (mean)	Pop Dens	1	0.068	0.268	78.566
Deaths/1 M/month 1 (max)	Pop Dens	1	0.135	0.111	125.948
Deaths/1 M log (Total)	Pop Dens	1	0.268	0.011	85.768
Deaths/day/1 M log (mean)	Pop Dens	1	0.240	0.018	82.137
Deaths/day/1 M log (median)	Pop Dens	1	0.269	0.016	73.783
Deaths/day/1 M log (max)	Pop Dens	1	0.117	0.110	82.663
Deaths/1 M/week 3 log (Total)	Pop Dens	1	0.083	0.194	86.441
Deaths/1 M/week 3 log (median)	Pop Dens	1	0.030	0.477	76.215
Deaths/1 M/week 3 log (mean)	Pop Dens	1	0.083	0.194	86.441
Deaths/1 M/week 3 log (max)	Pop Dens	1	0.049	0.320	81.729
Deaths/1 M/month 1 log (Total)	Pop Dens	1	0.158	0.083	75.431
Deaths/1 M/month 1 log (median)	Pop Dens	1	0.097	0.224	64.370
Deaths/1 M/month 1 log (mean)	Pop Dens	1	0.158	0.083	75.431
Deaths/1 M/month 1 log (max)	Pop Dens	1	0.192	0.053	68.063
Deaths/1 M (Total)	Urban (%)	1	0.021	0.509	295.303
Days to 0.1 Death/1 M	Urban (%)	1	0.019	0.534	144.380
Days to 1 Death/1 M	Urban (%)	1	0.021	0.511	167.266
Deaths/day/1 M (mean)	Urban (%)	1	0.029	0.436	110.639
Deaths/day/1 M (median)	Urban (%)	1	0.001	0.918	112.844
Deaths/day/1 M (max)	Urban (%)	1	0.108	0.126	156.878

Deaths/1 M/week 3 (Total)	Urban (%)	1	0.004	0.782	166.327
Deaths/1 M/week 3 (median)	Urban (%)	1	0.011	0.646	74.338
Deaths/1 M/week 3 (mean)	Urban (%)	1	0.004	0.782	80.707
Deaths/1 M/week 3 (max)	Urban (%)	1	0.001	0.879	107.527
Deaths/1 M/month 1 (Total)	Urban (%)	1	0.013	0.635	215.758
Deaths/1 M/month 1 (median)	Urban (%)	1	0.002	0.867	53.334
Deaths/1 M/month 1 (mean)	Urban (%)	1	0.013	0.635	79.710
Deaths/1 M/month 1 (max)	Urban (%)	1	0.019	0.564	128.476
Deaths/1 M log (Total)	Urban (%)	1	0.029	0.435	92.259
Deaths/day/1 M log (mean)	Urban (%)	1	0.022	0.499	87.924
Deaths/day/1 M log (median)	Urban (%)	1	0.012	0.637	80.122
Deaths/day/1 M log (max)	Urban (%)	1	0.059	0.262	84.126
Deaths/1 M/week 3 log (Total)	Urban (%)	1	0.000	0.965	88.345
Deaths/1 M/week 3 log (median)	Urban (%)	1	0.038	0.423	76.059
Deaths/1 M/week 3 log (mean)	Urban (%)	1	0.000	0.965	88.345
Deaths/1 M/week 3 log (max)	Urban (%)	1	0.000	0.974	82.844
Deaths/1 M/month 1 log (Total)	Urban (%)	1	0.006	0.736	78.732
Deaths/1 M/month 1 log (median)	Urban (%)	1	0.055	0.364	65.137
Deaths/1 M/month 1 log (mean)	Urban (%)	1	0.006	0.736	78.732
Deaths/1 M/month 1 log (max)	Urban (%)	1	0.014	0.621	72.057
Deaths/1 M (Total)	HDI	1	0.036	0.389	294.962
Days to 0.1 Death/1 M	HDI	1	0.012	0.614	144.529
Days to 1 Death/1 M	HDI	1	0.021	0.510	167.264
Deaths/day/1 M (mean)	HDI	1	0.052	0.295	110.092
Deaths/day/1 M (median)	HDI	1	0.020	0.521	112.393
Deaths/day/1 M (max)	HDI	1	0.110	0.122	156.822
Deaths/1 M/week 3 (Total)	HDI	1	0.026	0.473	165.834
Deaths/1 M/week 3 (median)	HDI	1	0.059	0.275	73.229
Deaths/1 M/week 3 (mean)	HDI	1	0.026	0.473	80.214
Deaths/1 M/week 3 (max)	HDI	1	0.016	0.570	107.189
Deaths/1 M/month 1 (Total)	HDI	1	0.036	0.425	215.290
Deaths/1 M/month 1 (median)	HDI	1	0.030	0.464	52.754
Deaths/1 M/month 1 (mean)	HDI	1	0.036	0.425	79.242
Deaths/1 M/month 1 (max)	HDI	1	0.056	0.317	127.711
Deaths/1 M log (Total)	HDI	1	0.149	0.069	89.229
Deaths/day/1 M log (mean)	HDI	1	0.154	0.064	84.592
Deaths/day/1 M log (median)	HDI	1	0.149	0.084	76.979
Deaths/day/1 M log (max)	HDI	1	0.223	0.023	79.745
Deaths/1 M/week 3 log (Total)	HDI	1	0.078	0.209	86.566
Deaths/1 M/week 3 log (median)	HDI	1	0.139	0.116	73.959
Deaths/1 M/week 3 log (mean)	HDI	1	0.078	0.209	86.566
Deaths/1 M/week 3 log (max)	HDI	1	0.079	0.206	81.038
Deaths/1 M/month 1 log (Total)	HDI	1	0.129	0.120	76.100

Deaths/1 M/month 1 log (median)	HDI	1	0.185	0.085	62.630
Deaths/1 M/month 1 log (mean)	HDI	1	0.129	0.120	76.100
Deaths/1 M/month 1 log (max)	HDI	1	0.168	0.072	68.651
Deaths/1 M (Total)	>65 yrs	1	0.042	0.348	294.807
Days to 0.1 Death/1 M	>65 yrs	1	0.099	0.143	142.409
Days to 1 Death/1 M	>65 yrs	1	0.275	0.010	160.369
Deaths/day/1 M (mean)	>65 yrs	1	0.039	0.366	110.404
Deaths/day/1 M (median)	>65 yrs	1	0.075	0.207	111.068
Deaths/day/1 M (max)	>65 yrs	1	0.021	0.508	159.012
Deaths/1 M/week 3 (Total)	>65 yrs	1	0.065	0.251	164.930
Deaths/1 M/week 3 (median)	>65 yrs	1	0.096	0.162	72.366
Deaths/1 M/week 3 (mean)	>65 yrs	1	0.065	0.251	79.310
Deaths/1 M/week 3 (max)	>65 yrs	1	0.054	0.296	106.322
Deaths/1 M/month 1 (Total)	>65 yrs	1	0.040	0.396	215.192
Deaths/1 M/month 1 (median)	>65 yrs	1	0.051	0.341	52.329
Deaths/1 M/month 1 (mean)	>65 yrs	1	0.040	0.396	79.144
Deaths/1 M/month 1 (max)	>65 yrs	1	0.037	0.417	128.103
Deaths/1 M log (Total)	>65 yrs	1	0.090	0.166	90.785
Deaths/day/1 M log (mean)	>65 yrs	1	0.111	0.121	85.738
Deaths/day/1 M log (median)	>65 yrs	1	0.135	0.102	77.337
Deaths/day/1 M log (max)	>65 yrs	1	0.094	0.154	83.259
Deaths/1 M/week 3 log (Total)	>65 yrs	1	0.211	0.031	83.127
Deaths/1 M/week 3 log (median)	>65 yrs	1	0.195	0.058	72.673
Deaths/1 M/week 3 log (mean)	>65 yrs	1	0.211	0.031	83.127
Deaths/1 M/week 3 log (max)	>65 yrs	1	0.200	0.037	77.942
Deaths/1 M/month 1 log (Total)	>65 yrs	1	0.159	0.082	75.405
Deaths/1 M/month 1 log (median)	>65 yrs	1	0.283	0.028	60.459
Deaths/1 M/month 1 log (mean)	>65 yrs	1	0.159	0.082	75.405
Deaths/1 M/month 1 log (max)	>65 yrs	1	0.106	0.161	70.092

Supplementary Table S2. ANOVA analyses of effect of BCG vaccination policy on COVID-19 mortality. Analyses included the United States as a single country (**Coarse analysis (United States as a Country)**) and by state (**Coarse analysis (United States as states)**), because some of its states are larger than many European, Asian, and Latin American countries. Analyses were conducted globally for all the countries for which data were available and also for a subset of countries after controlling for confounding variables (**Refined analysis (Controlling Confounding Variables)**). Mortality values are based on original numbers by country and corrected by the country's population and to the same time of the epidemic to allow standardized evaluations. Significant analysis ($p<0.05$) are denoted in red. **DF**: degrees of freedom, **R^2**: correlation coefficient, **AIC**: Akaike's information criteria. BCG Policy included current, interrupted and without ever (never) having a BCG national vaccination policy.

Dependent Variable	Independent Variable	F-statistic	DF	DF Residual	R^2	P-Value	AIC
**Coarse analysis (United States as states)**							
Deaths/1 M (Total)	BCG Policy	22.652	2	226	0.167	0.000	2851.683
Days to 0.1 Death/1 M	BCG Policy	9.237	2	192	0.088	0.000	1322.556
Days to 1 Death/1 M	BCG Policy	12.062	2	155	0.135	0.000	1157.465
Deaths/day/1 M (mean)	BCG Policy	21.469	2	196	0.180	0.000	1007.019
Deaths/day/1 M (median)	BCG Policy	20.426	2	196	0.172	0.000	869.126
Deaths/day/1 M (max)	BCG Policy	11.928	2	196	0.109	0.000	1725.294
Deaths/1 M/week 3 (Total)	BCG Policy	7.968	2	175	0.083	0.000	1744.938
Deaths/1 M/week 3 (median)	BCG Policy	17.817	2	175	0.169	0.000	756.657
Deaths/1 M/week 3 (mean)	BCG Policy	7.973	2	175	0.084	0.000	1052.179
Deaths/1 M/week 3 (max)	BCG Policy	4.158	2	175	0.045	0.017	1456.096
Deaths/1 M/month 1 (Total)	BCG Policy	10.964	2	122	0.152	0.000	1492.531
Deaths/1 M/month 1 (median)	BCG Policy	10.345	2	122	0.145	0.000	497.684
Deaths/1 M/month 1 (mean)	BCG Policy	11.000	2	122	0.153	0.000	642.132
Deaths/1 M/month 1 (max)	BCG Policy	7.456	2	122	0.109	0.001	1071.674
Deaths/1 M log (Total)	BCG Policy	89.762	2	196	0.478	0.000	762.574
Deaths/day/1 M log (mean)	BCG Policy	93.492	2	196	0.488	0.000	737.834
Deaths/day/1 M log (median)	BCG Policy	44.116	2	115	0.434	0.000	403.854
Deaths/day/1 M log (max)	BCG Policy	86.155	2	196	0.468	0.000	703.512
Deaths/1 M/week 3 log (Total)	BCG Policy	56.390	2	156	0.420	0.000	601.238
Deaths/1 M/week 3 log (median)	BCG Policy	41.757	2	113	0.425	0.000	395.097
Deaths/1 M/week 3 log (mean)	BCG Policy	58.068	2	156	0.427	0.000	598.349
Deaths/1 M/week 3 log (max)	BCG Policy	57.291	2	156	0.423	0.000	566.974
Deaths/1 M/month 1 log (Total)	BCG Policy	48.282	2	122	0.442	0.000	482.989
Deaths/1 M/month 1 log (median)	BCG Policy	36.943	2	91	0.448	0.000	315.617
Deaths/1 M/month 1 log (mean)	BCG Policy	49.584	2	122	0.448	0.000	481.252
Deaths/1 M/month 1 log (max)	BCG Policy	57.158	2	122	0.484	0.000	441.411
**Coarse analysis (United States as a Country)**							
Deaths/1 M (Total)	BCG Policy	70.017	2	171	0.450	0.000	2062.967
Days to 0.1 Death/1 M	BCG Policy	0.996	2	138	0.014	0.372	1003.696
Days to 1 Death/1 M	BCG Policy	0.731	2	101	0.014	0.484	798.832
Deaths/day/1 M (mean)	BCG Policy	48.473	2	142	0.406	0.000	642.772

Deaths/day/1 M (median)	BCG Policy	34.440	2	142	0.327	0.000	540.982
Deaths/day/1 M (max)	BCG Policy	26.807	2	142	0.274	0.000	1186.493
Deaths/1 M/week 3 (Total)	BCG Policy	16.246	2	123	0.209	0.000	1240.111
Deaths/1 M/week 3 (median)	BCG Policy	18.259	2	123	0.229	0.000	419.580
Deaths/1 M/week 3 (mean)	BCG Policy	16.246	2	123	0.209	0.000	749.742
Deaths/1 M/week 3 (max)	BCG Policy	13.032	2	123	0.175	0.000	1051.030
Deaths/1 M/month 1 (Total)	BCG Policy	15.362	2	86	0.263	0.000	1051.928
Deaths/1 M/month 1 (median)	BCG Policy	8.482	2	86	0.165	0.000	315.511
Deaths/1 M/month 1 (mean)	BCG Policy	15.362	2	86	0.263	0.000	446.515
Deaths/1 M/month 1 (max)	BCG Policy	13.808	2	86	0.243	0.000	766.657
Deaths/1 M log (Total)	BCG Policy	42.163	2	142	0.373	0.000	580.523
Deaths/day/1 M log (mean)	BCG Policy	38.175	2	142	0.350	0.000	565.486
Deaths/day/1 M log (median)	BCG Policy	25.786	2	70	0.424	0.000	264.525
Deaths/day/1 M log (max)	BCG Policy	34.349	2	142	0.326	0.000	534.755
Deaths/1 M/week 3 log (Total)	BCG Policy	19.941	2	106	0.273	0.000	435.423
Deaths/1 M/week 3 log (median)	BCG Policy	13.673	2	69	0.284	0.000	266.631
Deaths/1 M/week 3 log (mean)	BCG Policy	19.941	2	106	0.273	0.000	435.423
Deaths/1 M/week 3 log (max)	BCG Policy	18.334	2	106	0.257	0.000	415.150
Deaths/1 M/month 1 log (Total)	BCG Policy	21.196	2	86	0.330	0.000	363.809
Deaths/1 M/month 1 log (median)	BCG Policy	17.080	2	58	0.371	0.000	217.874
Deaths/1 M/month 1 log (mean)	BCG Policy	21.196	2	86	0.330	0.000	363.809
Deaths/1 M/month 1 log (max)	BCG Policy	23.228	2	86	0.351	0.000	333.817
**Refined analysis (Controlling Confounding Variables)**							
Deaths/1 M (Total)	BCG Policy	4.921	2	20	0.330	0.018	288.589
Days to 0.1 Death/1 M	BCG Policy	0.703	2	20	0.066	0.507	145.251
Days to 1 Death/1 M	BCG Policy	0.239	2	20	0.023	0.790	169.207
Deaths/day/1 M (mean)	BCG Policy	4.495	2	20	0.310	0.024	104.781
Deaths/day/1 M (median)	BCG Policy	3.037	2	20	0.233	0.070	108.756
Deaths/day/1 M (max)	BCG Policy	5.877	2	20	0.370	0.010	150.870
Deaths/1 M/week 3 (Total)	BCG Policy	1.411	2	19	0.129	0.268	165.367
Deaths/1 M/week 3 (median)	BCG Policy	1.997	2	19	0.174	0.163	72.379
Deaths/1 M/week 3 (mean)	BCG Policy	1.411	2	19	0.129	0.268	79.746
Deaths/1 M/week 3 (max)	BCG Policy	0.896	2	19	0.086	0.425	107.570
Deaths/1 M/month 1 (Total)	BCG Policy	1.718	2	17	0.168	0.209	214.335
Deaths/1 M/month 1 (median)	BCG Policy	2.270	2	17	0.211	0.134	50.631
Deaths/1 M/month 1 (mean)	BCG Policy	1.718	2	17	0.168	0.209	78.287
Deaths/1 M/month 1 (max)	BCG Policy	1.931	2	17	0.185	0.176	126.763
Deaths/1 M log (Total)	BCG Policy	6.317	2	20	0.387	0.007	83.680
Deaths/day/1 M log (mean)	BCG Policy	5.323	2	20	0.347	0.014	80.620
Deaths/day/1 M log (median)	BCG Policy	5.105	2	18	0.362	0.018	72.939
Deaths/day/1 M log (max)	BCG Policy	5.722	2	20	0.364	0.011	77.128
Deaths/1 M/week 3 log (Total)	BCG Policy	0.640	2	19	0.063	0.538	88.913
Deaths/1 M/week 3 log (median)	BCG Policy	1.398	2	16	0.149	0.276	75.737

Deaths/1 M/week 3 log (mean)	BCG Policy	0.640	2	19	0.063	0.538	88.913
Deaths/1 M/week 3 log (max)	BCG Policy	0.526	2	19	0.052	0.599	83.660
Deaths/1 M/month 1 log (Total)	BCG Policy	1.925	2	17	0.185	0.176	76.779
Deaths/1 M/month 1 log (median)	BCG Policy	5.243	2	14	0.428	0.020	58.600
Deaths/1 M/month 1 log (mean)	BCG Policy	1.925	2	17	0.185	0.176	76.779
Deaths/1 M/month 1 log (max)	BCG Policy	2.599	2	17	0.234	0.104	69.000

Supplementary Table S3. T-test analyses of effect of BCG vaccination policy on COVID-19 mortality. Analyses included the United States as a single country (**Coarse analysis (United States as a Country)**) and by state (**Coarse analysis (United States as states)**), because some of its states are larger than many European, Asian, and Latin American countries. Analyses were conducted globally for all the countries for which data were available and for a subset of countries after controlling for confounding variables (**Refined analysis (Controlling Confounding Variables)**). Mortality values are based on original numbers by country and corrected by the country's population and by the time of the epidemic to allow standardized evaluations. Significant analysis ($p<0.05$) are denoted in red. **DF**: degrees of freedom, **R^2**: correlation coefficient, **AIC**: Akaike's information criteria. BCG Policy included current, interrupted and without ever (never) having a BCG national vaccination policy.

Dependent Variable	Independent Variable	t-statistic	DF	P-value
**Coarse analysis (United States as states)**				
Deaths/1 M (Total)	BCG Current vs. Interrupted + Never	6.939	236.000	0.000
Days to 0.1 Death/1 M	BCG Current vs. Interrupted + Never	-3.976	198.000	1.000
Days to 1 Death/1 M	BCG Current vs. Interrupted + Never	-4.042	160.000	1.000
Deaths/day/1 M (mean)	BCG Current vs. Interrupted + Never	6.662	203.000	0.000
Deaths/day/1 M (median)	BCG Current vs. Interrupted + Never	6.545	203.000	0.000
Deaths/day/1 M (max)	BCG Current vs. Interrupted + Never	4.671	203.000	0.000
Deaths/1 M/week 3 (Total)	BCG Current vs. Interrupted + Never	3.931	179.000	0.000
Deaths/1 M/week 3 (median)	BCG Current vs. Interrupted + Never	6.084	179.000	0.000
Deaths/1 M/week 3 (mean)	BCG Current vs. Interrupted + Never	3.932	179.000	0.000
Deaths/1 M/week 3 (max)	BCG Current vs. Interrupted + Never	2.757	179.000	0.003
Deaths/1 M/month 1 (Total)	BCG Current vs. Interrupted + Never	4.674	125.000	0.000
Deaths/1 M/month 1 (median)	BCG Current vs. Interrupted + Never	4.647	125.000	0.000
Deaths/1 M/month 1 (mean)	BCG Current vs. Interrupted + Never	4.681	125.000	0.000
Deaths/1 M/month 1 (max)	BCG Current vs. Interrupted + Never	3.617	125.000	0.000
Deaths/1 M log (Total)	BCG Current vs. Interrupted + Never	13.195	203.000	0.000
Deaths/day/1 M log (mean)	BCG Current vs. Interrupted + Never	13.281	203.000	0.000
Deaths/day/1 M log (median)	BCG Current vs. Interrupted + Never	9.553	117.000	0.000
Deaths/day/1 M log (max)	BCG Current vs. Interrupted + Never	12.386	203.000	0.000
Deaths/1 M/week 3 log (Total)	BCG Current vs. Interrupted + Never	10.274	160.000	0.000
Deaths/1 M/week 3 log (median)	BCG Current vs. Interrupted + Never	9.148	114.000	0.000
Deaths/1 M/week 3 log (mean)	BCG Current vs. Interrupted + Never	10.415	160.000	0.000
Deaths/1 M/week 3 log (max)	BCG Current vs. Interrupted + Never	10.017	160.000	0.000
Deaths/1 M/month 1 log (Total)	BCG Current vs. Interrupted + Never	9.554	125.000	0.000
Deaths/1 M/month 1 log (median)	BCG Current vs. Interrupted + Never	8.641	92.000	0.000
Deaths/1 M/month 1 log (mean)	BCG Current vs. Interrupted + Never	9.674	125.000	0.000
Deaths/1 M/month 1 log (max)	BCG Current vs. Interrupted + Never	10.097	125.000	0.000
Deaths/1 M (Total)	BCG Current vs. Never	6.401	216.000	0.000
Days to 0.1 Death/1 M	BCG Current vs. Never	-4.029	178.000	1.000
Days to 1 Death/1 M	BCG Current vs. Never	-4.445	140.000	1.000

Deaths/day/1 M (mean)	BCG Current vs. Never	6.425	183.000	0.000
Deaths/day/1 M (median)	BCG Current vs. Never	6.201	183.000	0.000
Deaths/day/1 M (max)	BCG Current vs. Never	4.662	183.000	0.000
Deaths/1 M/week 3 (Total)	BCG Current vs. Never	3.876	159.000	0.000
Deaths/1 M/week 3 (median)	BCG Current vs. Never	6.063	159.000	0.000
Deaths/1 M/week 3 (mean)	BCG Current vs. Never	3.877	159.000	0.000
Deaths/1 M/week 3 (max)	BCG Current vs. Never	2.775	159.000	0.003
Deaths/1 M/month 1 (Total)	BCG Current vs. Never	4.700	107.000	0.000
Deaths/1 M/month 1 (median)	BCG Current vs. Never	4.958	107.000	0.000
Deaths/1 M/month 1 (mean)	BCG Current vs. Never	4.710	107.000	0.000
Deaths/1 M/month 1 (max)	BCG Current vs. Never	3.681	107.000	0.000
Deaths/1 M log (Total)	BCG Current vs. Never	11.923	183.000	0.000
Deaths/day/1 M log (mean)	BCG Current vs. Never	12.169	183.000	0.000
Deaths/day/1 M log (median)	BCG Current vs. Never	8.730	99.000	0.000
Deaths/day/1 M log (max)	BCG Current vs. Never	11.562	183.000	0.000
Deaths/1 M/week 3 log (Total)	BCG Current vs. Never	10.025	140.000	0.000
Deaths/1 M/week 3 log (median)	BCG Current vs. Never	8.936	97.000	0.000
Deaths/1 M/week 3 log (mean)	BCG Current vs. Never	10.197	140.000	0.000
Deaths/1 M/week 3 log (max)	BCG Current vs. Never	9.977	140.000	0.000
Deaths/1 M/month 1 log (Total)	BCG Current vs. Never	8.828	107.000	0.000
Deaths/1 M/month 1 log (median)	BCG Current vs. Never	7.649	76.000	0.000
Deaths/1 M/month 1 log (mean)	BCG Current vs. Never	8.972	107.000	0.000
Deaths/1 M/month 1 log (max)	BCG Current vs. Never	9.645	107.000	0.000
**Coarse analysis (United States as a Country)**				
Deaths/1 M (Total)	BCG Current vs. Interrupted + Never	8.669	181	0.000
Days to 0.1 Death/1 M	BCG Current vs. Interrupted + Never	-1.325	144	0.906
Days to 1 Death/1 M	BCG Current vs. Interrupted + Never	-0.952	106	0.828
Deaths/day/1 M (mean)	BCG Current vs. Interrupted + Never	7.637	149	0.000
Deaths/day/1 M (median)	BCG Current vs. Interrupted + Never	7.991	149	0.000
Deaths/day/1 M (max)	BCG Current vs. Interrupted + Never	4.706	149	0.000
Deaths/1 M/week 3 (Total)	BCG Current vs. Interrupted + Never	3.708	127	0.000
Deaths/1 M/week 3 (median)	BCG Current vs. Interrupted + Never	6.169	127	0.000
Deaths/1 M/week 3 (mean)	BCG Current vs. Interrupted + Never	3.708	127	0.000
Deaths/1 M/week 3 (max)	BCG Current vs. Interrupted + Never	2.886	127	0.002
Deaths/1 M/month 1 (Total)	BCG Current vs. Interrupted + Never	4.449	89	0.000
Deaths/1 M/month 1 (median)	BCG Current vs. Interrupted + Never	4.142	89	0.000
Deaths/1 M/month 1 (mean)	BCG Current vs. Interrupted + Never	4.449	89	0.000
Deaths/1 M/month 1 (max)	BCG Current vs. Interrupted + Never	3.308	89	0.001
Deaths/1 M log (Total)	BCG Current vs. Interrupted + Never	8.836	149	0.000
Deaths/day/1 M log (mean)	BCG Current vs. Interrupted + Never	8.338	149	0.000
Deaths/day/1 M log (median)	BCG Current vs. Interrupted + Never	7.060	72	0.000
Deaths/day/1 M log (max)	BCG Current vs. Interrupted + Never	7.600	149	0.000
Deaths/1 M/week 3 log (Total)	BCG Current vs. Interrupted + Never	6.064	110	0.000

Deaths/1 M/week 3 log (median)	BCG Current vs. Interrupted + Never	5.233	70	0.000
Deaths/1 M/week 3 log (mean)	BCG Current vs. Interrupted + Never	6.064	110	0.000
Deaths/1 M/week 3 log (max)	BCG Current vs. Interrupted + Never	5.601	110	0.000
Deaths/1 M/month 1 log (Total)	BCG Current vs. Interrupted + Never	6.290	89	0.000
Deaths/1 M/month 1 log (median)	BCG Current vs. Interrupted + Never	5.851	59	0.000
Deaths/1 M/month 1 log (mean)	BCG Current vs. Interrupted + Never	6.290	89	0.000
Deaths/1 M/month 1 log (max)	BCG Current vs. Interrupted + Never	6.365	89	0.000
Deaths/1 M (Total)	BCG Current vs. Never	12.607	161	0.000
Days to 0.1 Death/1 M	BCG Current vs. Never	-0.658	124	0.744
Days to 1 Death/1 M	BCG Current vs. Never	-0.396	86	0.653
Deaths/day/1 M (mean)	BCG Current vs. Never	10.813	129	0.000
Deaths/day/1 M (median)	BCG Current vs. Never	9.790	129	0.000
Deaths/day/1 M (max)	BCG Current vs. Never	6.982	129	0.000
Deaths/1 M/week 3 (Total)	BCG Current vs. Never	5.479	107	0.000
Deaths/1 M/week 3 (median)	BCG Current vs. Never	6.268	107	0.000
Deaths/1 M/week 3 (mean)	BCG Current vs. Never	5.479	107	0.000
Deaths/1 M/week 3 (max)	BCG Current vs. Never	4.856	107	0.000
Deaths/1 M/month 1 (Total)	BCG Current vs. Never	5.689	71	0.000
Deaths/1 M/month 1 (median)	BCG Current vs. Never	4.816	71	0.000
Deaths/1 M/month 1 (mean)	BCG Current vs. Never	5.689	71	0.000
Deaths/1 M/month 1 (max)	BCG Current vs. Never	4.926	71	0.000
Deaths/1 M log (Total)	BCG Current vs. Never	5.734	129	0.000
Deaths/day/1 M log (mean)	BCG Current vs. Never	5.266	129	0.000
Deaths/day/1 M log (median)	BCG Current vs. Never	4.662	54	0.000
Deaths/day/1 M log (max)	BCG Current vs. Never	5.093	129	0.000
Deaths/1 M/week 3 log (Total)	BCG Current vs. Never	3.975	90	0.000
Deaths/1 M/week 3 log (median)	BCG Current vs. Never	2.416	53	0.010
Deaths/1 M/week 3 log (mean)	BCG Current vs. Never	3.975	90	0.000
Deaths/1 M/week 3 log (max)	BCG Current vs. Never	3.896	90	0.000
Deaths/1 M/month 1 log (Total)	BCG Current vs. Never	3.974	71	0.000
Deaths/1 M/month 1 log (median)	BCG Current vs. Never	2.393	43	0.011
Deaths/1 M/month 1 log (mean)	BCG Current vs. Never	3.974	71	0.000
Deaths/1 M/month 1 log (max)	BCG Current vs. Never	4.362	71	0.000
**Refined analysis (Controlling Confounding Variables)**				
Deaths/1 M (Total)	BCG Current vs. Interrupted + Never	2.586	21	0.009
Days to 0.1 Death/1 M	BCG Current vs. Interrupted + Never	0.911	21	0.186
Days to 1 Death/1 M	BCG Current vs. Interrupted + Never	0.266	21	0.396
Deaths/day/1 M (mean)	BCG Current vs. Interrupted + Never	2.718	21	0.006
Deaths/day/1 M (median)	BCG Current vs. Interrupted + Never	2.081	21	0.025
Deaths/day/1 M (max)	BCG Current vs. Interrupted + Never	3.122	21	0.003
Deaths/1 M/week 3 (Total)	BCG Current vs. Interrupted + Never	1.607	20	0.062
Deaths/1 M/week 3 (median)	BCG Current vs. Interrupted + Never	1.933	20	0.034
Deaths/1 M/week 3 (mean)	BCG Current vs. Interrupted + Never	1.607	20	0.062

Deaths/1 M/week 3 (max)	BCG Current vs. Interrupted + Never	1.308	20	0.103
Deaths/1 M/month 1 (Total)	BCG Current vs. Interrupted + Never	1.900	18	0.037
Deaths/1 M/month 1 (median)	BCG Current vs. Interrupted + Never	1.876	18	0.038
Deaths/1 M/month 1 (mean)	BCG Current vs. Interrupted + Never	1.900	18	0.037
Deaths/1 M/month 1 (max)	BCG Current vs. Interrupted + Never	1.962	18	0.033
Deaths/1 M log (Total)	BCG Current vs. Interrupted + Never	3.219	21	0.002
Deaths/day/1 M log (mean)	BCG Current vs. Interrupted + Never	3.035	21	0.003
Deaths/day/1 M log (median)	BCG Current vs. Interrupted + Never	3.128	19	0.003
Deaths/day/1 M log (max)	BCG Current vs. Interrupted + Never	3.154	21	0.002
Deaths/1 M/week 3 log (Total)	BCG Current vs. Interrupted + Never	1.098	20	0.143
Deaths/1 M/week 3 log (median)	BCG Current vs. Interrupted + Never	1.501	17	0.076
Deaths/1 M/week 3 log (mean)	BCG Current vs. Interrupted + Never	1.098	20	0.143
Deaths/1 M/week 3 log (max)	BCG Current vs. Interrupted + Never	1.023	20	0.159
Deaths/1 M/month 1 log (Total)	BCG Current vs. Interrupted + Never	2.017	18	0.029
Deaths/1 M/month 1 log (median)	BCG Current vs. Interrupted + Never	2.388	15	0.015
Deaths/1 M/month 1 log (mean)	BCG Current vs. Interrupted + Never	2.017	18	0.029
Deaths/1 M/month 1 log (max)	BCG Current vs. Interrupted + Never	2.305	18	0.017
Deaths/1 M (Total)	BCG Current vs. Never	4.808	8	0.001
Days to 0.1 Death/1 M	BCG Current vs. Never	1.325	8	0.111
Days to 1 Death/1 M	BCG Current vs. Never	0.719	8	0.246
Deaths/day/1 M (mean)	BCG Current vs. Never	4.997	8	0.001
Deaths/day/1 M (median)	BCG Current vs. Never	2.912	8	0.010
Deaths/day/1 M (max)	BCG Current vs. Never	12.536	8	0.000
Deaths/1 M/week 3 (Total)	BCG Current vs. Never	1.076	7	0.159
Deaths/1 M/week 3 (median)	BCG Current vs. Never	1.302	7	0.117
Deaths/1 M/week 3 (mean)	BCG Current vs. Never	1.076	7	0.159
Deaths/1 M/week 3 (max)	BCG Current vs. Never	0.887	7	0.202
Deaths/1 M/month 1 (Total)	BCG Current vs. Never	2.120	6	0.039
Deaths/1 M/month 1 (median)	BCG Current vs. Never	1.100	6	0.157
Deaths/1 M/month 1 (mean)	BCG Current vs. Never	2.120	6	0.039
Deaths/1 M/month 1 (max)	BCG Current vs. Never	2.208	6	0.035
Deaths/1 M log (Total)	BCG Current vs. Never	5.284	8	0.000
Deaths/day/1 M log (mean)	BCG Current vs. Never	4.294	8	0.001
Deaths/day/1 M log (median)	BCG Current vs. Never	3.306	7	0.007
Deaths/day/1 M log (max)	BCG Current vs. Never	3.804	8	0.003
Deaths/1 M/week 3 log (Total)	BCG Current vs. Never	0.412	7	0.346
Deaths/1 M/week 3 log (median)	BCG Current vs. Never	0.316	6	0.381
Deaths/1 M/week 3 log (mean)	BCG Current vs. Never	0.412	7	0.346
Deaths/1 M/week 3 log (max)	BCG Current vs. Never	0.438	7	0.337
Deaths/1 M/month 1 log (Total)	BCG Current vs. Never	1.843	6	0.057
Deaths/1 M/month 1 log (median)	BCG Current vs. Never	0.132	5	0.450
Deaths/1 M/month 1 log (mean)	BCG Current vs. Never	1.843	6	0.057
Deaths/1 M/month 1 log (max)	BCG Current vs. Never	2.132	6	0.039

Supplementary Table S4. Correlation analyses of between BCG vaccination coverage (%) and COVID-19 mortality. Analyses included the United States as a single country (**Coarse analysis (United States as a Country)**) and by state (**Coarse analysis (United States as states)**), because some of its states are larger than many European, Asian, and Latin American countries. Analyses were conducted globally for all the countries for which data were available and for a subset of countries after controlling for confounding variables (**Refined analysis (Controlling Confounding Variables)**). Mortality values are based on original numbers by country and corrected by the country's population and by the time of the epidemic to allow standardized evaluations. Significant analysis ($p<0.05$) are denoted in red. **DF**: degrees of freedom, R^2: correlation coefficient, **AIC**: Akaike's information criteria.

Dependent Variable	Independent Variable	DF	R^2	P-Value	AIC
**Coarse analysis (United States as states)**					
Deaths/1 M (Total)	BCG % (mean)	1	0.136	0.000	2881.170
Days to 0.1 Death/1 M	BCG % (mean)	1	0.030	0.015	1338.341
Days to 1 Death/1 M	BCG % (mean)	1	0.115	0.000	1158.996
Deaths/day/1 M (mean)	BCG % (mean)	1	0.155	0.000	1019.258
Deaths/day/1 M (median)	BCG % (mean)	1	0.143	0.000	881.125
Deaths/day/1 M (max)	BCG % (mean)	1	0.093	0.000	1742.152
Deaths/1 M/week 3 (Total)	BCG % (mean)	1	0.076	0.000	1744.446
Deaths/1 M/week 3 (median)	BCG % (mean)	1	0.145	0.000	759.792
Deaths/1 M/week 3 (mean)	BCG % (mean)	1	0.076	0.000	1051.687
Deaths/1 M/week 3 (max)	BCG % (mean)	1	0.041	0.007	1454.902
Deaths/1 M/month 1 (Total)	BCG % (mean)	1	0.156	0.000	1489.923
Deaths/1 M/month 1 (median)	BCG % (mean)	1	0.136	0.000	496.991
Deaths/1 M/month 1 (mean)	BCG % (mean)	1	0.157	0.000	639.521
Deaths/1 M/month 1 (max)	BCG % (mean)	1	0.111	0.000	1069.332
Deaths/1 M log (Total)	BCG % (mean)	1	0.262	0.000	841.162
Deaths/day/1 M log (mean)	BCG % (mean)	1	0.275	0.000	813.656
Deaths/day/1 M log (median)	BCG % (mean)	1	0.303	0.000	430.004
Deaths/day/1 M log (max)	BCG % (mean)	1	0.295	0.000	766.106
Deaths/1 M/week 3 log (Total)	BCG % (mean)	1	0.260	0.000	637.962
Deaths/1 M/week 3 log (median)	BCG % (mean)	1	0.306	0.000	414.852
Deaths/1 M/week 3 log (mean)	BCG % (mean)	1	0.265	0.000	635.884
Deaths/1 M/week 3 log (max)	BCG % (mean)	1	0.284	0.000	599.357
Deaths/1 M/month 1 log (Total)	BCG % (mean)	1	0.242	0.000	519.190
Deaths/1 M/month 1 log (median)	BCG % (mean)	1	0.282	0.000	338.344
Deaths/1 M/month 1 log (mean)	BCG % (mean)	1	0.247	0.000	518.093
Deaths/1 M/month 1 log (max)	BCG % (mean)	1	0.313	0.000	475.172
Deaths/1 M (Total)	BCG % (median)	1	0.141	0.000	2879.926
Days to 0.1 Death/1 M	BCG % (median)	1	0.036	0.008	1337.187
Days to 1 Death/1 M	BCG % (median)	1	0.118	0.000	1158.504

Deaths/day/1 M (mean)	BCG % (median)	1	0.160	0.000	1018.121
Deaths/day/1 M (median)	BCG % (median)	1	0.146	0.000	880.414
Deaths/day/1 M (max)	BCG % (median)	1	0.095	0.000	1741.721
Deaths/1 M/week 3 (Total)	BCG % (median)	1	0.076	0.000	1744.317
Deaths/1 M/week 3 (median)	BCG % (median)	1	0.147	0.000	759.382
Deaths/1 M/week 3 (mean)	BCG % (median)	1	0.076	0.000	1051.559
Deaths/1 M/week 3 (max)	BCG % (median)	1	0.041	0.006	1454.817
Deaths/1 M/month 1 (Total)	BCG % (median)	1	0.157	0.000	1489.771
Deaths/1 M/month 1 (median)	BCG % (median)	1	0.137	0.000	496.896
Deaths/1 M/month 1 (mean)	BCG % (median)	1	0.158	0.000	639.369
Deaths/1 M/month 1 (max)	BCG % (median)	1	0.112	0.000	1069.203
Deaths/1 M log (Total)	BCG % (median)	1	0.279	0.000	836.601
Deaths/day/1 M log (mean)	BCG % (median)	1	0.293	0.000	808.786
Deaths/day/1 M log (median)	BCG % (median)	1	0.325	0.000	426.176
Deaths/day/1 M log (max)	BCG % (median)	1	0.314	0.000	760.581
Deaths/1 M/week 3 log (Total)	BCG % (median)	1	0.280	0.000	633.507
Deaths/1 M/week 3 log (median)	BCG % (median)	1	0.326	0.000	411.507
Deaths/1 M/week 3 log (mean)	BCG % (median)	1	0.286	0.000	631.338
Deaths/1 M/week 3 log (max)	BCG % (median)	1	0.304	0.000	594.819
Deaths/1 M/month 1 log (Total)	BCG % (median)	1	0.262	0.000	515.869
Deaths/1 M/month 1 log (median)	BCG % (median)	1	0.300	0.000	335.915
Deaths/1 M/month 1 log (mean)	BCG % (median)	1	0.267	0.000	514.716
Deaths/1 M/month 1 log (max)	BCG % (median)	1	0.335	0.000	471.061
**Coarse analysis (United States as a Country)**					
Deaths/1 M (Total)	BCG % (mean)	1	0.237	0.000	2140.516
Days to 0.1 Death/1 M	BCG % (mean)	1	0.007	0.332	1008.942
Days to 1 Death/1 M	BCG % (mean)	1	0.007	0.411	797.634
Deaths/day/1 M (mean)	BCG % (mean)	1	0.242	0.000	683.583
Deaths/day/1 M (median)	BCG % (mean)	1	0.236	0.000	563.032
Deaths/day/1 M (max)	BCG % (mean)	1	0.126	0.000	1226.192
Deaths/1 M/week 3 (Total)	BCG % (mean)	1	0.108	0.000	1253.196
Deaths/1 M/week 3 (median)	BCG % (mean)	1	0.147	0.000	430.238
Deaths/1 M/week 3 (mean)	BCG % (mean)	1	0.108	0.000	762.827
Deaths/1 M/week 3 (max)	BCG % (mean)	1	0.077	0.002	1063.203
Deaths/1 M/month 1 (Total)	BCG % (mean)	1	0.234	0.000	1053.432
Deaths/1 M/month 1 (median)	BCG % (mean)	1	0.141	0.000	316.041
Deaths/1 M/month 1 (mean)	BCG % (mean)	1	0.234	0.000	448.019
Deaths/1 M/month 1 (max)	BCG % (mean)	1	0.166	0.000	773.300
Deaths/1 M log (Total)	BCG % (mean)	1	0.068	0.001	646.415
Deaths/day/1 M log (mean)	BCG % (mean)	1	0.059	0.003	625.025
Deaths/day/1 M log (median)	BCG % (mean)	1	0.225	0.000	287.457
Deaths/day/1 M log (max)	BCG % (mean)	1	0.078	0.001	586.033
Deaths/1 M/week 3 log (Total)	BCG % (mean)	1	0.057	0.012	461.798

Deaths/1 M/week 3 log (median)	BCG % (mean)	1	0.100	0.007	281.064
Deaths/1 M/week 3 log (mean)	BCG % (mean)	1	0.057	0.012	461.798
Deaths/1 M/week 3 log (max)	BCG % (mean)	1	0.070	0.006	437.666
Deaths/1 M/month 1 log (Total)	BCG % (mean)	1	0.071	0.012	390.912
Deaths/1 M/month 1 log (median)	BCG % (mean)	1	0.123	0.006	236.128
Deaths/1 M/month 1 log (mean)	BCG % (mean)	1	0.071	0.012	390.912
Deaths/1 M/month 1 log (max)	BCG % (mean)	1	0.115	0.001	359.421
Deaths/1 M (Total)	BCG % (median)	1	0.248	0.000	2137.799
Days to 0.1 Death/1 M	BCG % (median)	1	0.003	0.524	1009.488
Days to 1 Death/1 M	BCG % (median)	1	0.008	0.364	797.484
Deaths/day/1 M (mean)	BCG % (median)	1	0.254	0.000	681.140
Deaths/day/1 M (median)	BCG % (median)	1	0.245	0.000	561.389
Deaths/day/1 M (max)	BCG % (median)	1	0.130	0.000	1225.417
Deaths/1 M/week 3 (Total)	BCG % (median)	1	0.109	0.000	1253.100
Deaths/1 M/week 3 (median)	BCG % (median)	1	0.154	0.000	429.309
Deaths/1 M/week 3 (mean)	BCG % (median)	1	0.109	0.000	762.731
Deaths/1 M/week 3 (max)	BCG % (median)	1	0.077	0.002	1063.147
Deaths/1 M/month 1 (Total)	BCG % (median)	1	0.233	0.000	1053.449
Deaths/1 M/month 1 (median)	BCG % (median)	1	0.142	0.000	315.910
Deaths/1 M/month 1 (mean)	BCG % (median)	1	0.233	0.000	448.036
Deaths/1 M/month 1 (max)	BCG % (median)	1	0.166	0.000	773.312
Deaths/1 M log (Total)	BCG % (median)	1	0.086	0.000	643.613
Deaths/day/1 M log (mean)	BCG % (median)	1	0.077	0.001	622.291
Deaths/day/1 M log (median)	BCG % (median)	1	0.264	0.000	283.668
Deaths/day/1 M log (max)	BCG % (median)	1	0.099	0.000	582.599
Deaths/1 M/week 3 log (Total)	BCG % (median)	1	0.079	0.003	459.265
Deaths/1 M/week 3 log (median)	BCG % (median)	1	0.127	0.002	278.926
Deaths/1 M/week 3 log (mean)	BCG % (median)	1	0.079	0.003	459.265
Deaths/1 M/week 3 log (max)	BCG % (median)	1	0.092	0.001	435.031
Deaths/1 M/month 1 log (Total)	BCG % (median)	1	0.092	0.004	388.837
Deaths/1 M/month 1 log (median)	BCG % (median)	1	0.148	0.002	234.327
Deaths/1 M/month 1 log (mean)	BCG % (median)	1	0.092	0.004	388.837
Deaths/1 M/month 1 log (max)	BCG % (median)	1	0.142	0.000	356.659
**Refined analysis (Controlling Confounding Variables)**					
Deaths/1 M (Total)	BCG % (mean)	1	0.232	0.020	289.735
Days to 0.1 Death/1 M	BCG % (mean)	1	0.049	0.310	143.658
Days to 1 Death/1 M	BCG % (mean)	1	0.082	0.185	165.776
Deaths/day/1 M (mean)	BCG % (mean)	1	0.243	0.017	104.910
Deaths/day/1 M (median)	BCG % (mean)	1	0.221	0.024	107.107
Deaths/day/1 M (max)	BCG % (mean)	1	0.221	0.023	153.750
Deaths/1 M/week 3 (Total)	BCG % (mean)	1	0.061	0.269	165.035
Deaths/1 M/week 3 (median)	BCG % (mean)	1	0.059	0.276	73.237
Deaths/1 M/week 3 (mean)	BCG % (mean)	1	0.061	0.269	79.415

Deaths/1 M/week 3 (max)	BCG % (mean)	1	0.069	0.238	105.984
Deaths/1 M/month 1 (Total)	BCG % (mean)	1	0.182	0.061	211.998
Deaths/1 M/month 1 (median)	BCG % (mean)	1	0.037	0.418	52.615
Deaths/1 M/month 1 (mean)	BCG % (mean)	1	0.182	0.061	75.950
Deaths/1 M/month 1 (max)	BCG % (mean)	1	0.275	0.018	122.430
Deaths/1 M log (Total)	BCG % (mean)	1	0.149	0.069	89.229
Deaths/day/1 M log (mean)	BCG % (mean)	1	0.116	0.112	85.608
Deaths/day/1 M log (median)	BCG % (mean)	1	0.146	0.087	77.057
Deaths/day/1 M log (max)	BCG % (mean)	1	0.125	0.098	82.470
Deaths/1 M/week 3 log (Total)	BCG % (mean)	1	0.007	0.717	88.199
Deaths/1 M/week 3 log (median)	BCG % (mean)	1	0.043	0.392	75.953
Deaths/1 M/week 3 log (mean)	BCG % (mean)	1	0.007	0.717	88.199
Deaths/1 M/week 3 log (max)	BCG % (mean)	1	0.013	0.618	82.564
Deaths/1 M/month 1 log (Total)	BCG % (mean)	1	0.079	0.230	77.216
Deaths/1 M/month 1 log (median)	BCG % (mean)	1	0.014	0.646	65.856
Deaths/1 M/month 1 log (mean)	BCG % (mean)	1	0.079	0.230	77.216
Deaths/1 M/month 1 log (max)	BCG % (mean)	1	0.170	0.071	68.608
Deaths/1 M (Total)	BCG % (median)	1	0.259	0.013	288.890
Days to 0.1 Death/1 M	BCG % (median)	1	0.052	0.297	143.597
Days to 1 Death/1 M	BCG % (median)	1	0.079	0.193	165.853
Deaths/day/1 M (mean)	BCG % (median)	1	0.277	0.010	103.862
Deaths/day/1 M (median)	BCG % (median)	1	0.228	0.021	106.918
Deaths/day/1 M (max)	BCG % (median)	1	0.250	0.015	152.900
Deaths/1 M/week 3 (Total)	BCG % (median)	1	0.056	0.289	165.144
Deaths/1 M/week 3 (median)	BCG % (median)	1	0.055	0.292	73.324
Deaths/1 M/week 3 (mean)	BCG % (median)	1	0.056	0.289	79.524
Deaths/1 M/week 3 (max)	BCG % (median)	1	0.063	0.260	106.125
Deaths/1 M/month 1 (Total)	BCG % (median)	1	0.183	0.060	211.971
Deaths/1 M/month 1 (median)	BCG % (median)	1	0.032	0.450	52.714
Deaths/1 M/month 1 (mean)	BCG % (median)	1	0.183	0.060	75.923
Deaths/1 M/month 1 (max)	BCG % (median)	1	0.303	0.012	121.638
Deaths/1 M log (Total)	BCG % (median)	1	0.175	0.047	88.514
Deaths/day/1 M log (mean)	BCG % (median)	1	0.139	0.079	84.985
Deaths/day/1 M log (median)	BCG % (median)	1	0.162	0.071	76.668
Deaths/day/1 M log (max)	BCG % (median)	1	0.147	0.071	81.884
Deaths/1 M/week 3 log (Total)	BCG % (median)	1	0.009	0.682	88.157
Deaths/1 M/week 3 log (median)	BCG % (median)	1	0.045	0.386	75.932
Deaths/1 M/week 3 log (mean)	BCG % (median)	1	0.009	0.682	88.157
Deaths/1 M/week 3 log (max)	BCG % (median)	1	0.014	0.598	82.531
Deaths/1 M/month 1 log (Total)	BCG % (median)	1	0.090	0.198	76.968
Deaths/1 M/month 1 log (median)	BCG % (median)	1	0.017	0.617	65.810
Deaths/1 M/month 1 log (mean)	BCG % (median)	1	0.090	0.198	76.968
Deaths/1 M/month 1 log (max)	BCG % (median)	1	0.198	0.049	67.927
